# Analgesic efficacy of postoperative bilateral, ultrasound-guided, posterior transversus abdominis plane block for laparoscopic colorectal cancer surgery: a randomized, prospective, controlled study

**DOI:** 10.1186/s12871-021-01317-6

**Published:** 2021-04-06

**Authors:** Yang Zhao, Han-Ying Zhang, Zong-Yi Yuan, Yi Han, Yi-Rong Chen, Qi-lin Liu, Tao Zhu

**Affiliations:** 1 Department of Anesthesiology, Affiliated Hospital of North Sichuan Medical College, Nanchong, Sichuan China; 2 Department of Anesthesiology, Pidu District People’s Hospital, 156# East Street, Pitong Town, Pidu District, Chengdu, Sichuan 611730 People’s Republic of China; 3 Departments of Oral and Maxillofacial, Nanchong Central Hospital, The Second Clinical Medical College, North Sichuan Medical College (University), Nanchong, Sichuan China; 4 Department of Anesthesiology, Affiliated Hospital of Southwest Medical University, Luzhou, Sichuan China; 5 Department of Anesthesiology, West China Hospital of Sichuan University, Chengdu, 610041 Sichuan P. R. China

**Keywords:** TAP block, Colorectal cancer surgery, Analgesia technique, Ropivacaine

## Abstract

**Background:**

We assessed whether a postoperative bilateral, ultrasound-guided, posterior transversus abdominis plane (TAP) block could reduce 24 h rescue tramadol requirement compared with placebo in patients undergoing elective laparoscopic colorectal cancer surgery.

**Methods:**

Patients scheduled to undergo elective laparoscopic surgery following the diagnosis of colorectal cancer were included in this study and randomized into Group and Group Control. The patients received a postoperative bilateral, ultrasound-guided, posterior TAP block in either 20 mL of 0.5% ropivacaine (Group TAP) per side or an equivalent volume of normal saline (Group Control). The primary outcome was the cumulative consumption of rescue tramadol within 24 h after the surgery. Secondary endpoints included (1) resting and movement numerical rating scale (NRS) pain scores at 2, 4, 6, 12, 24, 48, and 72 h; (2) incidences of related side effects; (3) time to the first request for rescue tramadol; (4) patient satisfaction regarding postoperative analgesia; (5) time to restoration of intestinal function; (6) time to mobilization; and (7) the length of hospital stay.

**Results:**

In total, 92 patients were randomized, and 82 patients completed the analysis. The total rescue tramadol requirement (median [interquartile range]) within the first 24 h was lower in Group TAP (0 [0, 87.5] mg) than in Group Control (100 [100, 200] mg), *P* < 0.001. The posterior TAP block reduced resting and movement NRS pain scores at 2, 4, 6, 12, and 24 h after surgery (all *P* < 0.001) but showed similar scores at 48 h or 72 h. A higher level of satisfaction with postoperative analgesia was observed in Group TAP on day 1 (*P* = 0.002), which was similar on days 2 (*P* = 0.702) and 3 (*P* = 0.551), compared with the Group Control. A few incidences of opioid-related side effects (P < 0.001) and a lower percentage of patients requiring rescue tramadol analgesia within 24 h (P < 0.001) were observed in Group TAP. The time to the first request for rescue analgesia was prolonged, and the time to mobilization and flatus was reduced with a shorter hospital stay in Group TAP as compared with Group Control.

**Conclusions:**

A postoperative bilateral, ultrasound-guided, posterior TAP block resulted in better pain management and a faster recovery in patients undergoing laparoscopic colorectal cancer surgery, without adverse effects.

**Trial registration:**

The study was registered at http://www.chictr.org.cn (ChiCTR-IPR-17012650; Sep 12, 2017).

## Background

Perioperative analgesia is essential for patients undergoing elective colorectal surgery in the enhanced recovery after surgery (ERAS) program. However, postoperative pain associated with colorectal surgery is considered neuropathic and requires a multimodal treatment approach to achieve effective pain control with fewer side effects [1, 2]. Transversus abdominis plane (TAP) block was suggested as a necessary part of the analgesia approach to control postoperative pain in several abdominal and gynecological surgical procedures [3]. The TAP technique includes injecting local anesthetics into a plane between the internal oblique (IO) and transversus abdominis (TA) muscles, which contain the thoracolumbar nerves originating from T6 to L1 spinal roots that supply the skin, muscles, parietal peritoneum, and sensation to the anterolateral abdominal wall [4, 5]. Performing an ultrasound-guided block enhanced the accuracy and efficacy of injecting local anesthetics into the TAP [6, 7].

The three primary approaches to TAP are subcostal, lateral, and posterior. The subcostal approach provides analgesia to the upper abdomen, whereas lateral and posterior approaches reduce the pain in the lower abdomen. Previous studies indicated that lateral TAP reduced the resting pain score within the first 6 h of laparoscopic colorectal surgery [8]. In contrast, posterior TAP provided 12 to 36 h of postoperative analgesia for total abdominal hysterectomy or cesarean delivery surgery [9, 10]. However, there is limited evidence suggesting that compared with systemic opioids or placebo, posterior TAP could reduce opioid consumption and pain scores after laparoscopic colorectal cancer surgery [2]. This study hypothesized that a postoperative bilateral, ultrasound-guided, posterior TAP could reduce the requirement for rescue analgesics within the first 24 h compared with placebo in patients undergoing laparoscopic colorectal cancer surgery.

## Methods

### Patients

This randomized, double-blinded, prospective clinical trial was registered with the Chinese registry of clinical trials at http://www.chictr.org.cn (ChiCTR-IPR-17012650; Sep 12, 2017). The Research Ethics Committee of the Affiliated Hospital of North Sichuan Medical College approved the study (approval no. 2017/049). This study adhered to the applicable CONSORT guidelines and was conducted from January 2018 to December 2019 in the Affiliated Hospital of North Sichuan Medical College. Informed written consent was obtained from all participants. Inclusion criteria were patients aged 18 to 65 years without previous abdominal surgery; American Society of Anesthesiologists classification (ASA) I–III; ability to express pain; and patients who were undergoing elective laparoscopic colorectal cancer surgery. Exclusion criteria were patients who had undergone any surgery again after the elective laparoscopic colorectal cancer surgery until discharged; a history of an allergic reaction to local anesthetics or opioids; weighing less than 45 kg (to reduce the risk of anesthetic toxicity); a history of recent exposure to opioids; body mass index (BMI) ≥ 30 kg/m^2^; exposure to pain medication 24 h before the surgery; inability to use patient-controlled intravenous analgesia; and patients undergoing resections requiring perineal incisions.

### Randomization and blinding

On the day of surgery, consented patients were randomly assigned to Group TAP or Group Control (1:1) using SPSS 25.0 software (Statistical Program for Social Sciences, SPSS Inc., Chicago, Illinois, USA) by Q.-L.L., who was not involved in other parts of the study.

The anonymity of allocation was ensured by enclosing assignments in sealed, opaque, and sequentially numbered envelopes opened by a nurse (Y.H.) only upon patient’s arrival in the operation room. The nurse prepared 0.5% ropivacaine or saline (40 mL) for all patients during the study period according to the allocation and did not participate in any other related process. The allocation was blinded for all patients, surgeons, anesthesiologists, and follow-up observers until the end of the study.

### Intervention

**Fig. 1:**
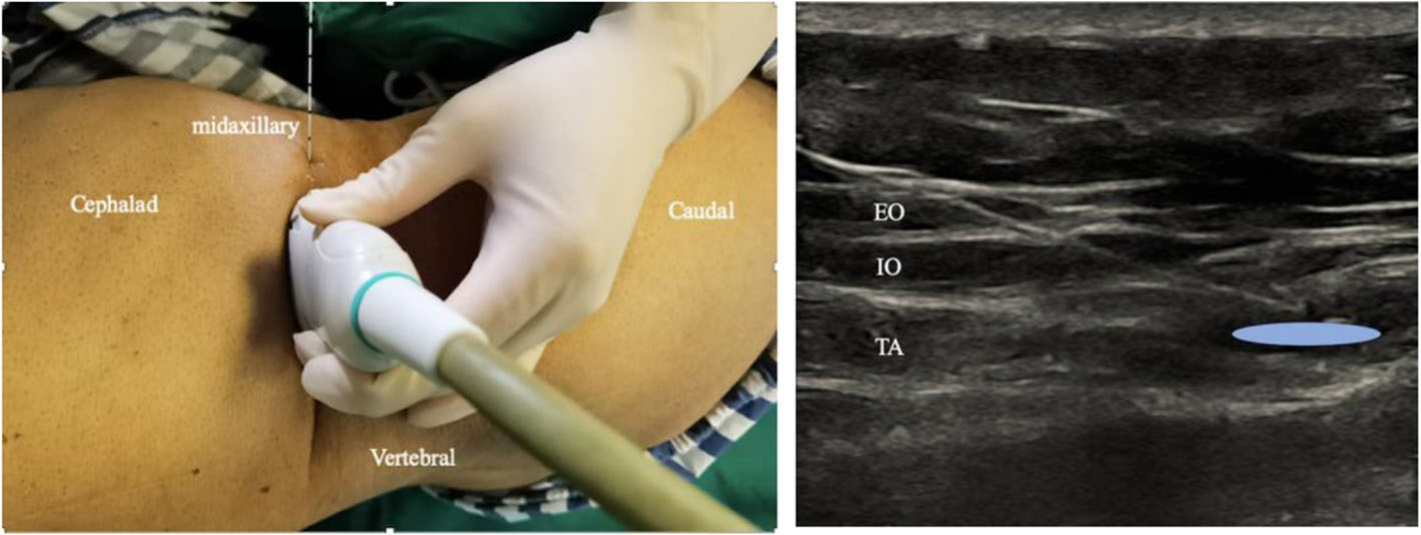
Posterior approach of transversus abdominis plane (TAP) block. Note: **a** The patient was kept in semi-lateral position, the probe position and needle trajectory were displayed. The probe is placed posterior to the midaxillary line between the costal margin and the iliac crest. The needle is inserted in plane. **b** Corresponding ultrasound images. Posterior approach located in the end of transversus abdominis plane where TAP transmigrate into aponeurosis. The injection site is at the TAP between internal oblique and transversus abdominis posterior to the midaxillary line and near the aponeurosis. White dashed line: needle trajectory. Light blue area: the deposition site of local anesthetic. TA: transversus abdominis; IO: internal oblique; EO: external oblique

Before extubation, all patients received ultrasound-guided bilateral posterior TAP block by an experienced anesthesiologist at the end of the surgery. The patient was kept in a semi-lateral position and received the posterior TAP block. An ultrasound probe was placed posterior to the mid-axillary line between the costal margin and the iliac crest [11] (Fig. 1). When scanning posteriorly, transversus abdominis tailed off and turned into aponeurosis. Subsequently, a blunt-ended needle was injected into the TAP between the internal oblique and transversus abdominis, posterior to the mid-axillary line and near the aponeurosis. Real-time imaging allowed the anesthesiologist to observe the passage of the needle through the internal oblique and its entry into the TAP endpoint near the aponeurosis. Whether the needle was placed correctly was confirmed by injecting the saline solution into the muscle plane and assessing the spread. After the confirmation, 40 mL of 0.5% ropivacaine was injected (20 mL per side) in Group TAP and an equivalent volume of saline in Group Control. The successful injection was defined as the appearance of a hypoechoic ellipsoid with well-defined margins on ultrasound imaging [12, 13].

### Anesthesia, surgery, and postoperative analgesia

All patients received standard perioperative care. Patients were routinely monitored by electrocardiography. In addition, non-invasive arterial blood pressure, arterial oxygen saturation, and end-tidal carbon dioxide were monitored, and patients were placed in the Trendelenburg position. General anesthesia was induced and maintained using intravenous midazolam (0.04 mg/kg), propofol (2.0–3.0 mg/kg), sufentanil (0.3 μg/kg) in both the groups. Endotracheal intubation was performed using IV administration of rocuronium (0.6 mg/kg). Sufentanil (10 μg) and rocuronium (10 mg) were administered intravenously before the incision. Anesthesia was maintained with a combined IV-inhaled anesthesia: sevoflurane (2–4%) with oxygen 2 L/min, rocuronium (0.1–0.2 mg/kg/h) was applied to maintain muscle relaxation, and remifentanil (0.1 μg/kg/min) was used to maintain intraoperative analgesia. Sevoflurane end-tidal concentrations were titrated to maintain the bispectral index value between 40 and 60 for all patients. An IV infusion of atropine and ephedrine was used to maintain blood pressure and heart rate at the preoperative baseline range (increase and decrease in the width did not exceed 20% of the baseline value). All patients were intravenously administered 0.15 μg/kg sufentanil 30 min before the surgery finished, following the patient-controlled intravenous anesthesia (PCIA) which was set locked until the patient becomes awake. The PCIA contained 100 μg of sufentanil and 98 mL of saline. The PCIA was set as follows: background infusion of 2 μg/h sufentanil, a bolus dose of 2 μg sufentanil, and a lockout interval of 5 min [14].

The postoperative intravenous antiemetic regimen consisted of dexamethasone (5 mg) administered at induction and ondansetron (4 mg) administered after the surgery. After the surgery, anesthesiologists performed ultrasound-guided bilateral posterior TAP block in all patients. After patients awoke, the tube was extubated, and they were transferred to the post-anesthesia care unit (PACU) for further monitoring. If the patient complained of an 11-point numerical rating scale for pain (NRS, 0 = no pain; 10 = worst pain imaginable) exceeded 3, a muscular injection of rescue tramadol (50 mg) was offered, which was allowed to repeat at a maximum dose of 400 mg per day [15]. In addition, rescue ﻿antiemetics were provided to patients complaining of nausea or vomiting. Early mobilization was encouraged since the patient was transferred to the ward. Patients met the discharge criteria if they could have a soft diet, were completely mobilized, and had an NRS score lower than 3.

### Follow-up and outcomes

Patients were evaluated from PACU until discharge from the hospital by the same investigator who was blinded to randomization. The primary outcome was the cumulative consumption of﻿ rescue tramadol within 24 h after the surgery. Secondary endpoints included (1) resting and movement NRS scores assessed at 2, 4, 6, 12, 24, 48, and 72 h, postoperatively [16–18]; (2) incidences of related side effects, such as nausea, vomiting, pruritus, sedation, and respiratory depression; (3) time to the first requirement of rescue tramadol muscular injection; (4) patient satisfaction on postoperative analgesia at 24, 48, and 72 h after the surgery using a 5-point scale [19] (1 = very unsatisfied, 2 = unsatisfied, 3 = fair, 4 = satisfied, and 5 = very satisfied); (5) time for restoration of intestinal function; (6) time to the first mobilization; and (7) length of hospital stay (number of nights spent in the hospital from the date of surgery to discharge).

### Sample size

The sample size was based on the 24 h rescue tramadol requirement of patients undergoing laparoscopic colorectal cancer surgery. To calculate the sample size, a clinically significant reduction in 24 h tramadol consumption was considered as a 20% absolute reduction with a conservative assumption. Based on initial pilot studies, we found that 24 h tramadol requirement was 110 ± 34.2 mg in the control group of 10 subjects. With a statistical power of 0.8 and a type 1 error rate of 0.05 to detect 20% improvement as conservative, the minimum requirement to demonstrate a difference using a two-tailed Student’s *t*-test was a sample size of 38 patients per group. Considering a possible dropout rate of 20%, we included 92 patients in this study.

### Statistical analysis

The collected data were analyzed using SPSS 25.0 software (Statistical Program for Social Sciences, SPSS Inc., Chicago, Illinois, USA), with a two-tailed *P*-value < 0.05 considered statistically significant. Continuous variables were presented as means ±standard deviations or medians ±interquartile range (IQR), or absolute numbers. The log-rank test was to compare the time to the first request of rescue tramadol. Categorical variables are presented as percentages. The two-sample Student’s *t*-test or the Mann–Whitney U-test was used for continuous variables, and the Chi-squared test compared differences in the qualitative data.

## Results

**Fig. 2:**
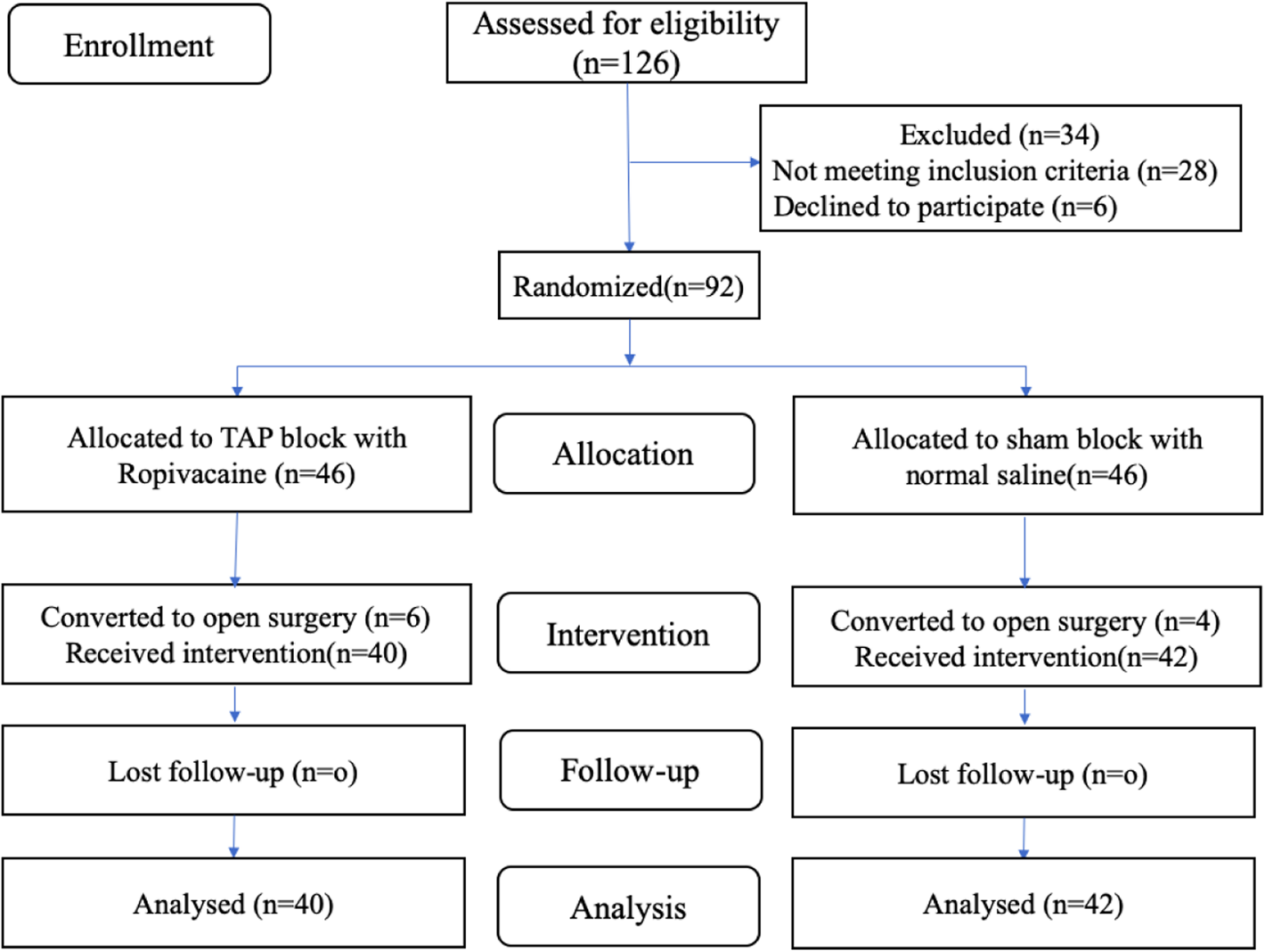
Consort flow study diagram. Note: TAP: transversus abdominis plane

One hundred twenty-six consecutive patients were assessed for eligibility between January 2018 and December 2019. Of these, 28 patients did not meet the inclusion criteria, and 6 patients refused to participate. The remaining 92 patients were randomized into Group TAP (*n* = 46) and Group Control (n = 46) to receive TAP intervention and a sham block, respectively. Laparoscopic surgeries were converted to open surgery in six patients of Group TAP and four patients in the Group Control. These ten patients were excluded from the outcome analysis. Eighty-two patients successfully underwent a postoperative bilateral, ultrasound-guided, posterior TAP block, which was determined by the bilateral appearance of a hypoechoic ellipsoid ultrasound image [12, 13]. Because no patient was lost to follow-up, 40 patients from Group TAP and 42 from Group Control were analyzed (Fig. 2). Both groups were similar in terms of sex, age, BMI, ASA, surgery duration, comorbidities, intraoperative analgesics, and extraction incision used (Table 1).

**Table 1:** Demographic and intraoperative characteristics

variables	Group TAP (*n* = 40)	Group Control (*n* = 42)	*P* value
Mean age, year	51.4 ± 7.4	52.1 ± 8.4	
Gender, male: female	18:22	22:20	
BMI (kg/m2)	23.6 ± 2.7	23.8 ± 2.6	
ASA I / II / III	8/28/4	7/33/2	0.903
Operation time (min)	162.7 ± 33.4	164.3 ± 30.4	0.820
Type of operation			0.995
Right hemicolectomy	16 (40)	16 (38)	/
Left hemicolectomy	12 (30)	13 (31)	/
Anterior resection	6 (15)	7 (17)	/
Sigmoid colectomy	6 (15)	6 (14)	/
Intraoperative sufentanil usage (ug)	37.7 ± 4.2	37.6 ± 4.3	0.941
Intraoperative remifentanil usage (mg)	1.0 ± 0.3	1.0 ± 0.3	0.979

Note: Data are presented as mean ± SD or the number of cases or no. (%) of patients. *ASA* American Society of Anesthesiologists, *BMI* body mass index, *Group TAP* transversus abdominis plane block

The (median [interquartile range]) cumulative consumption of rescue tramadol within 24 h was significantly lower in Group TAP (0 mg [0, 87.5]), compared with Group Control (100 mg [100, 200]), *P* < 0.001 (Table 2).

**Table 2:** Comparison of clinical outcomes between the groups

	Group TAP (n = 40)	Group Control (n = 42)	P value
Tramadol consumption within 24 h after surgery (mg) ^a^	0 (0, 87.5)	100 (100, 200)	< 0.001
Time to first requirement of rescue tramadol muscular injection (min)^a^	1440(285,1440.00)	50(30, 90)	< 0.001
time to flatus (h)	32.4 ± 6.2	39.0 ± 8.7	< 0.001
time to mobilization (h)	27.9 ± 7.8	33.9 ± 8.2	0.001
length of hospital stay (d)	3.4 ± 0.5	4.0 ± 0.6	< 0.001

Note: Data are presented as mean ± SD, unless otherwise indicated. ^a^Data are presented as median and quartiles, and analyzed by Mann-Whitney U test. Group TAP = transversus abdominis plane block

**Fig. 3:**
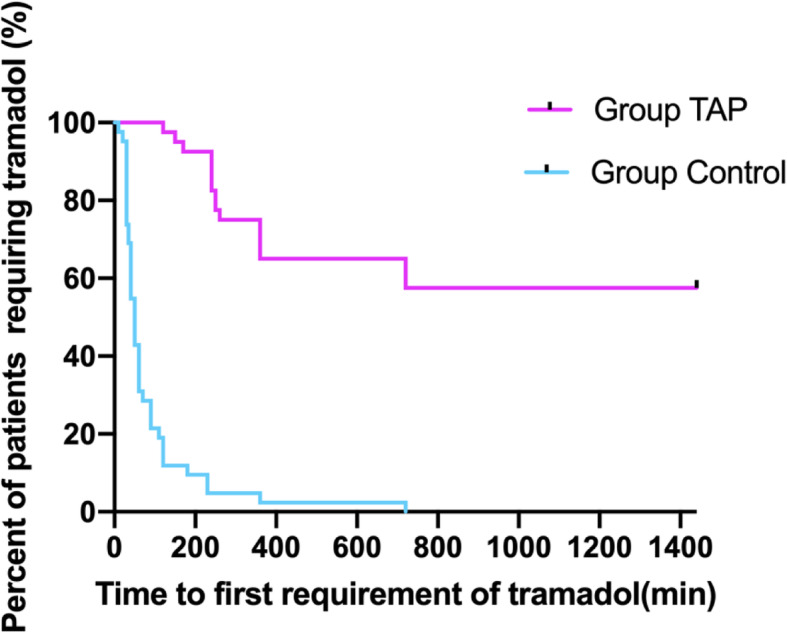
Kaplan-Meier curve depicting time to first tramadol requirement during postoperative 24-h follow-up among two groups. Note: Group TAP = transversus abdominis plane block, P<0.001

A longer time to first tramadol muscular injection request was observed in Group TAP than the Group Control (Fig. 3). The (median [interquartile range]) time to first request for tramadol was significantly more in Group TAP (1440 min [285, 1440]) compared with Group Control (50 min [30, 90]; *P* < 0.001) (Table 2).

**Fig. 4:**
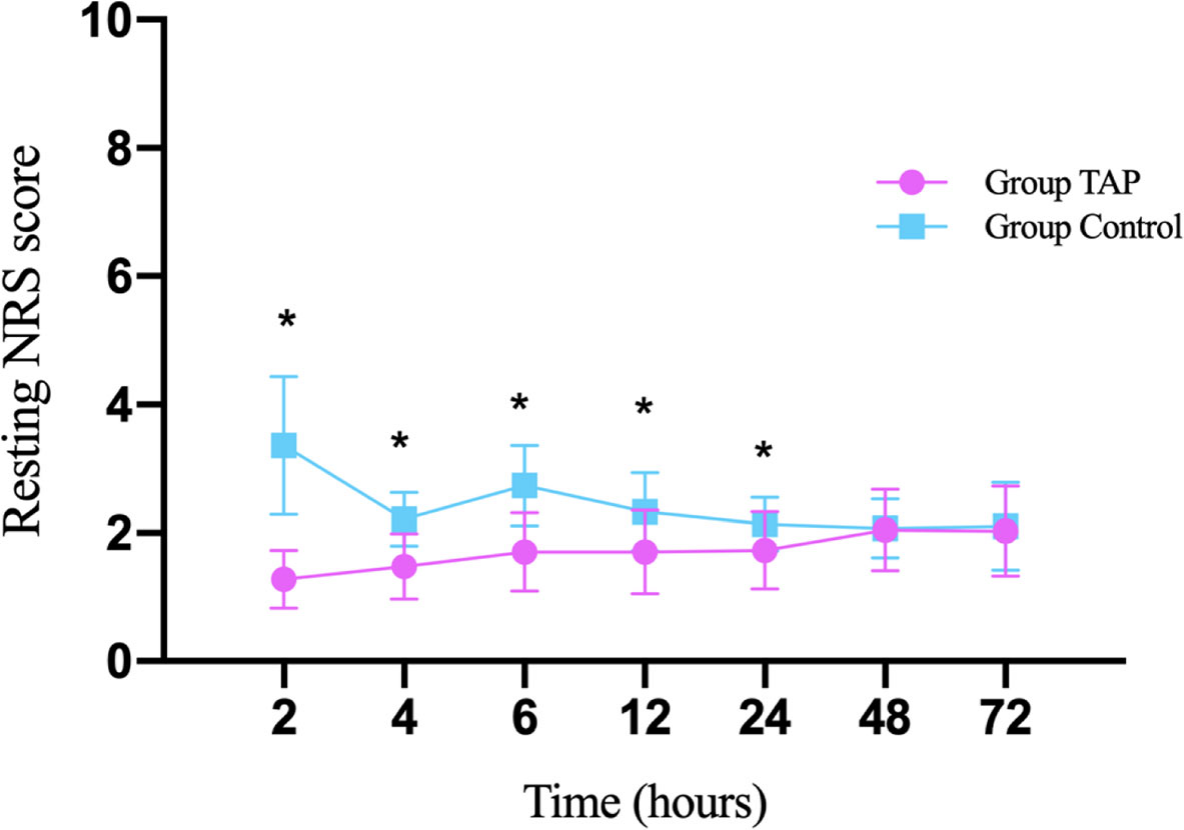
Comparison of resting NRS scores at different times after surgery between the groups. Note: Mean postoperative resting NRS scores assessed by using an 11-point numerical rating scale (0 = no pain and 10 = the worst imaginable pain) at different times after surgery in each group. *Indicates NRS score significantly difference (*P* < 0.001, t-test) between two groups. Group TAP = transversus abdominis plane block

**Fig. 5:**
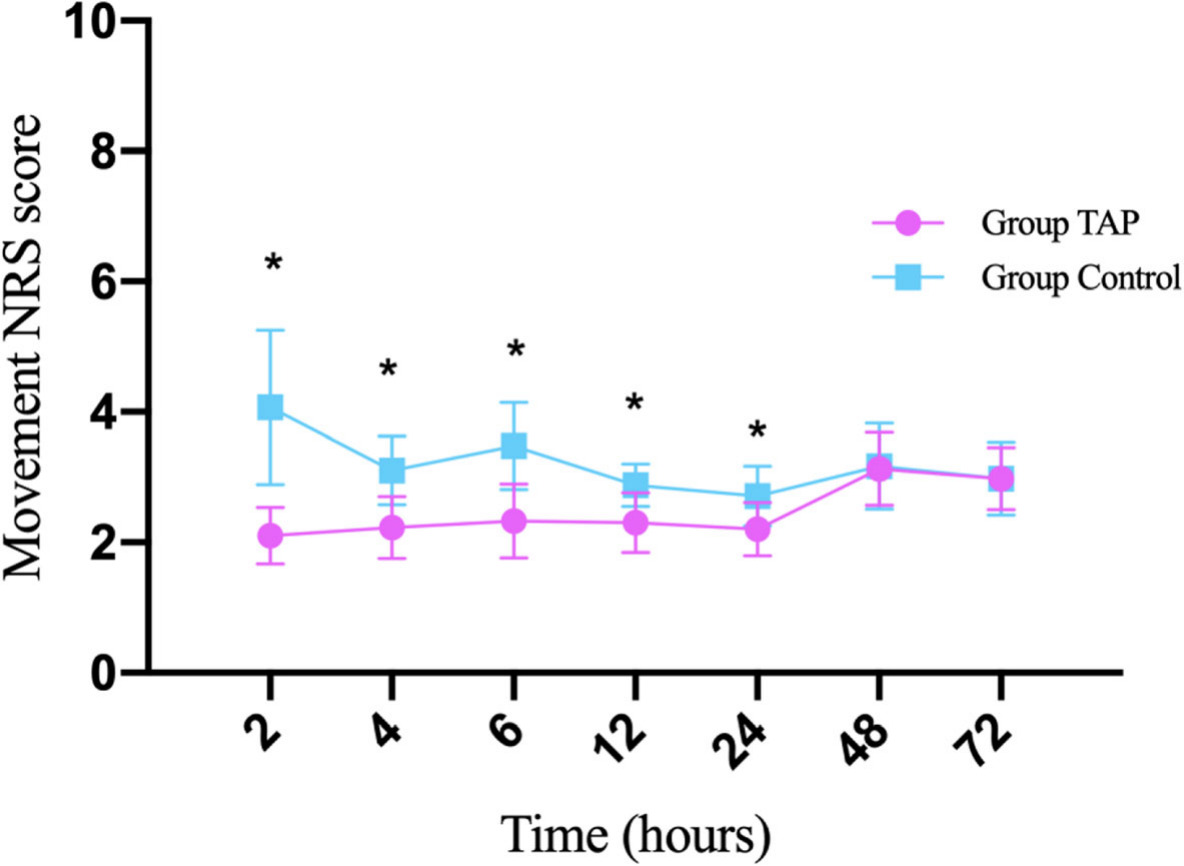
Comparison of movement NRS scores at different times after surgery between the groups. Note: Mean postoperative moving NRS scores assessed by using an 11-point numerical rating scale (0 = no pain and 10 = the worst imaginable pain) at different times after surgery in each group. *Indicates NRS score significantly difference (P < 0.001, t-test) between two groups. Group TAP = transversus abdominis plane block

﻿Resting NRS scores were lower in Group TAP than in Group Control at 2 (1.3 ± 0.5 vs. 3.4 ± 1.1), 4 (1.5 ± 0.5 vs. 2.2 ± 0.4), 6 (1.7 ± 0.6 vs. 2.7 ± 0.6), 12 (1.7 ± 0.7 vs. 2.3 ± 0.6), and 24 h (1.7 ± 0.6 vs. 2.1 ± 0.4) after the surgery (all P < 0.001) but similar at 48 (2.1 ± 0.6 vs. 2.1 ± 0.5, *P* = 0.862) or 72 h (2.0 ± 0.7 vs. 2.1 ± 0.7, *P* = 0.648). In addition, movement NRS scores were lower in Group TAP than in Group Control at 2 (2.1 ± 0.4 vs. 4.1 ± 1.2), 4 (2.2 ± 0.5 vs. 3.1 ± 0.5), 6 (2.3 ± 0.6 vs. 3.5 ± 0.7), 12 (2.3 ± 0.5 vs. 2.9 ± 0.3), and 24 h (2.2 ± 0.4 vs. 2.7 ± 0.5) after the surgery (all *P* < 0.001) but similar at 48 h (3.1 ± 0.6 vs. 3.2 ± 0.7, *P* = 0.760) or 72 h (3.0 ± 0.5 vs. 3.0 ± 0.6, *P* = 0.992) (Figs. 4 and 5).

The incidence of nausea and vomiting was lower in Group TAP than in Group Control. No pruritus, sedation, and respiratory depression occurred in both groups (Table 3).

**Table 3:** Comparison of postoperative side effects between the groups

Side effects	Group TAP (n = 40)	Group Control (n = 42)	P value
Nausea	11/40	32/42	<0.001
Vomiting	3/40	17/42	<0.001
Pruritus	0/40	0/42	/
Sedation	0/40	0/42	/
Respiratory depression	0/40	0/42	/

Note: Data are presented as the number of case. Group TAP = transversus abdominis plane block

Table 4 shows patients’ postoperative satisfaction level on analgesia. Patients’ satisfaction was significantly higher in Group TAP on postoperative day 1 (*P* = 0.012) but similar on days 2 and 3, compared with Group Control.

**Table 4:** Comparison of satisfaction on postoperative analgesia at different times between the groups

	Group TAP (n = 40)	Group Control (n = 42)	P value
24 h after surgery	4 (3,4)	3(3,4)	0.002
48 h after surgery	4(4,4)	4(4,4)	0.702
72 h after surgery	4(4,4)	4(3,4)	0.551

Note: Data are presented as median and quartiles, and analyzed by Mann-Whitney U test. Assessed satisfaction using a 5-point scale (1 = very unsatisfied, 2 = unsatisfied, 3 = fair, 4 = satisfied, and 5 = very satisfied). Group TAP = transversus abdominis plane block

The first time to get out of bed was significantly earlier in Group TAP than in Group Control (27.9 ± 7.8 vs. 33.9 ± 8.2 h, *P* = 0.001). Time to first passage of flatus was significantly earlier in Group TAP than in Group Control (32.4 ± 6.2 vs. 39.0 ± 8.7 h, *P* < 0.001) (Table 2). The mean length of hospital stay was significantly shorter in Group TAP than in Group Control (3.4 ± 0.5 vs. 3.9 ± 0.6 days, P < 0.001) (Table 2).

## Discussion

In this study, a postoperative bilateral, ultrasound-guided, posterior TAP using 0.5% ropivacaine (20 mL per side) reduced and delayed the requirement for rescue analgesics within the first 24 h. Besides, the resting and movement pain scores also decreased at 2, 4, 6, 12, and 24 h in Group TAP. Less related side effects, accelerated bowel function recovery, and shorter hospital stay were observed in Group TAP compared with Group Control.

Excessive perioperative opioid consumption increases the incidence of postoperative nausea and vomiting (PONV), sedation, pruritus, urinary retention, bowel dysfunction, and respiratory depression, and delays postoperative recovery [20–22]. The resting and movement NRS scores in Group TAP were lower than those in Group Control at 2, 4, 6, 12, and 24 h after the surgery. Although the pain scores in Group TAP differentiate from Group Control slightly, early effective analgesia contributed to lower and delayed rescue analgesia requirement of tramadol muscular injection in Group TAP, resulting in tramadol spare-effect. In this study, the application of posterior TAP in Group TAP significantly reduced tramadol consumption and the incidence of PONV 24 h after the surgery compared with the Group Control. Thus, the reduction in PONV could be explained by the decrease in tramadol-related adverse effects. Early pain relief and fewer side effects allowed the patients to get up and ambulate. Moreover, it was associated with earlier mobilization, better bowel function recovery, shorter hospital stay, and better satisfaction [23]. However, the pain scores in the two groups were similar at 48 or 72 h and were consistently very low, this could be due to the analgesic effects of PCIA and the minimal invasive advantages of laparoscopic over open surgery for all participants [24]. However, half-day reduction of hospital stay was statistically significant (3.4 ± 0.5 vs. 3.9 ± 0.6 d, *P* < 0.001), the clinical significance was relatively limited.

Postoperative analgesia is an essential part of perioperative anesthetic management and the enhanced recovery program. After the surgery, acute pain significantly contributes to the increased hospital stay and patient dissatisfaction [25]. Pain derived from the abdominal wall incision was the primary component experienced by the patients after abdominal surgery [12]. Recently, TAP has been recommended as an essential component of multimodal analgesia techniques because it provides effective analgesia for abdominal surgical procedures, including colorectal surgery [26, 27]. It blocks the T6–L1 spinal nerves’ neural branches dominating the anterolateral abdominal wall [11]. After the anterior rami of these nerves exit their respective vertebral foramina, they enter the anterior abdomen muscles and reach the neurofascial plane between the internal oblique and transversus abdominis muscles. The sensory nerve branch first sends out a lateral cutaneous branch in the mid-axillary line and continues to move within the plane to supply the anterior skin [12]. The posterior TAP is located between the costal margin and the iliac crest. In the present study, the needle was inserted at the mid-axillary line. A large volume of a local anesthetic was deposited in the transverse abdominal plane by the mid-axillary line puncture that blocked the lateral cutaneous branches, thereby completely blocking the anterior abdominal wall [12]. Similar research on cadavers and volunteers demonstrated that posterior TAP provided analgesia effect from the anterior–lateral abdominal area to the post-axillary line [28, 29]. In addition, different approaches of TAP substantially influenced the spread pattern of local anesthetics within the plane. It has been reported that local anesthetics mostly spread into the layer between the internal oblique and external oblique muscles in the subcostal and lateral TAP approaches without extending into the paravertebral space, resulting in somatic pain relief in the anterior abdominal wall. However, in posterior TAP, local anesthetic may partly enter the paravertebral space covering T4 to L1 in a retrograde fashion and potentially blocking a few degrees along with the thoracolumbar sympathetic system, resulting in a more comprehensive somatic pain relief in the abdominal wall [30–32]. Because the sympathetic nervous system mediates pain after the surgery, the posterior TAP could achieve a prolonged analgesic effect. Besides, the postoperative TAP may prolong the analgesic effect by delaying the metabolism process compared with preoperative TAP [33]. Finally, the posterior TAP injection probably causes deposition of the local anesthetic in the aponeurosis, which acts as “warehouse effect” that plausibly intensifies and prolongs the effect of posterior TAP [34, 35]. Taking all these into account, we considered that a single shot of posterior and postoperative TAP with 0.5% ropivacaine relieved the pain efficiently during 24 h follow-up although the duration of ropivacaine only lasted 4–8 h in pharmacokinetic, our results were similar to previous studies [12, 36].

A previous study indicated that a preoperative bilateral, ultrasound-guided, lateral TAP block using 2 mg/kg levobupivacaine (40 mL) equally split between the sides (up to a total maximum dose of 150 mg) decreased the pain scores only at 2, 4, and 6 h [33]. However, we observed that the postoperative bilateral, ultrasound-guided, posterior TAP produced 24 h analgesia in laparoscopic colorectal surgery, indicating that the posterior TAP prolonged postoperative analgesia. Thus, it is not surprising that the posterior TAP block in our study provided a reliable, satisfied, and durable analgesia effect for laparoscopic cancer surgery. Moreover, both preoperative and postoperative timings are suitable for performing TAP. However, we selected the postoperative TAP over preoperative TAP, owing to its advantages, such as avoiding local anesthetic distribution within the muscle layers caused by the long head-down position and delaying the local anesthetic metabolization [33].

TAP block is a superficial technique, where the target depth is usually between 1 and 3 cm, with a small but finite risk of complications [37]. The ultrasound-guided TAP block is a relatively safe intervention without adverse outcomes [12, 38, 39]. The semi-lateral position ensures abdominal organs to be considerably away from the needle trajectory, making posterior TAP safe. Therefore, ultrasound-guided posterior TAP block is considered a safe technique for postoperative analgesia.

Our study had several limitations. First, our study did not measure the plasma concentrations of ropivacaine. Ropivacaine for TAP block could result in systemic toxicity, indicating it is essential to monitor the local anesthetic concentration. However, any symptoms of local anesthetic systemic toxicity, such as seizures, cardiovascular collapse, metallic taste, and tinnitus, did not occur in our study, whether immediately after the block or in the follow-up period. Second, the sensory block effect of posterior TAP was not assessed because the patient was still under general anesthesia. However, we confirmed that the needle passed through different muscle layers until its tip entered the layer between the internal oblique and transverse muscles. A hypoechoic ellipsoid with well-defined margins was indicative of successful injection under the ultrasound-guided TAP block, similar to previous studies [12, 13]. Third, although TAP block was a considerable analgesic ﻿regimen for laparoscopic colorectal cancer surgery, it was not more beneficial in patients who underwent different abdominal surgeries, especially those who received multimodal analgesia, which contained epidural analgesia, continuous lidocaine infusion, wound local infiltration, or quadratus lumborum block [40–42].

## Conclusions

This prospective, randomized, double-blinded trial showed that postoperative bilateral, ultrasound-guided, posterior TAP block using 20 mL of 0.5% ropivacaine per side decreased the cumulative consumption of rescue tramadol within 24 h after the surgery. The pain scores also decreased significantly at rest and movement at 2, 4, 6, 12, and 24 h for patients undergoing laparoscopic colorectal cancer surgery. A delayed and lower requirement of tramadol muscular injection accelerated the bowel function recovery, and shorter hospital stay was observed compared with placebo without an increase in adverse events.

## Data Availability

Data have been uploaded successfully to the Chinese registry of clinical trials at http://www.chictr.org.cn. The datasets used and/or analyzed during the current study are available from the corresponding author upon reasonable request.

## Abbreviations

TAP: Transversus abdominis plane
IV: Intravenous
NRS: Numerical rating scale
ERAS: Enhanced recovery after surgery
IO: Internal oblique
TA: Transversus abdominis
ASA: American Society of Anesthesiologists classification
BMI: Body mass index
PCIA: Patient-controlled intravenous anesthesia
PACU: Post-anesthesia care unit
IQR: Interquartile range
PONV: Postoperative nausea and vomiting
